# Genotype-by-environment interaction in coast redwood outside natural distribution - search for environmental cues

**DOI:** 10.1186/s12863-020-0821-1

**Published:** 2020-02-10

**Authors:** Jaroslav Klápště, Dean Meason, Heidi S. Dungey, Emily J. Telfer, Paul Silcock, Simon Rapley

**Affiliations:** 1 Scion (New Zealand Forest Research Institute Ltd.), 49 Sala Street, Rotorua, 3010 New Zealand; 2 NZ Forestry Ltd., 701A Pollen Street, Thames, 3500 New Zealand; 3 The New Zealand Redwood Company, P.O. Box 1343, Taupō, 3351 New Zealand

**Keywords:** Climate change, Clonal forestry, Universal response function, Genotype x environment interaction, *Sequoia sempervirens*

## Abstract

**Background:**

Effective matching of genotypes and environments is required for the species to reach optimal productivity and act effectively for carbon sequestration. A common garden experiment across five different environments was undertaken to assess genotype x environment interaction (GxE) of coast redwood in order to understand the performance of genotypes across environments.

**Results:**

The quantitative genetic analysis discovered no GxE between investigated environments for diameter at breast height (DBH). However, no genetic component was detected at one environment possibly due to stressful conditions. The implementation of universal response function allowed for the identification of important environmental factors affecting species productivity. Additionally, this approach enabled us to predict the performance of species across the New Zealand environmental conditions.

**Conclusions:**

In combination with quantitative genetic analysis which identified genetically superior material, the URF model can directly identify the optimal geographical regions to maximize productivity. However, the finding of ideally uncorrelated climatic variables for species with narrow ecological amplitude is rather challenging, which complicates construction of informative URF model. This, along with a small number of tested environments, tended to overfit a prediction model which resulted in extreme predictions in untested environments.

## Background

Global climate change is expected to alter the net primary productivity of all terrestrial ecosystems over the next few decades. Understanding the effects on plant productivity, not only of the sequestration of carbon from elevated CO_2_ levels but also of interactions between carbon, nitrogen and water availability, is essential for the future sustainable management of natural resources [1]. Forest ecosystems have an important role in the global carbon cycle by storing carbon in the soil, live biomass, litter and deadwood. The amount of carbon stored is estimated to be around 45% of all terrestrial carbon [2]. The annual carbon uptake of forest ecosystems is approximately 2.3 Pg of C per year, which represents approximately 33% of anthropogenic carbon emissions from fossil fuels and changes in land use [2, 3].

Changes in climate observed globally over a number of years are expected to continue and are likely to affect the types of trees that can be grown in particular environments [4]. Effective management of genetic resources for sustainable productivity and carbon sequestration of forests requires a good understanding of the full range of species genetic variability over a wide range of environments. Common garden experiments can be used to compare genetically distinct strains, families or populations under identical environmental conditions [5] and to dissect genetic divergence and phenotypic plasticity [6]. A number of common garden studies on various species have found significant proportions of phenotypic variability in production traits (such as height or diameter) are caused by differences in climatic adaptation between populations along environmental gradients such as latitude [7–10] or altitude [11]. In the northern hemisphere, trees from southern/low elevation locations are consistently taller than trees from northern/high elevation areas, and these differences are associated with the number of frost-free days in the location of origin [7]. Therefore, planting populations from southern/low elevations in colder/higher environments may be required to compensate for global warming [12, 13] in order for species to remain effective production-wise and for carbon sequestration [14, 15]. Wang et al. [16] proposed universal response function as a combination of response and transfer function to capture these trends and make a well-informed decision about the optimal combination of genetic resources and growth conditions to maximise forest productivity under future predicted climate.

Coast redwood (*Sequoia sempervirens* (Lamb. Ex D. Don) Endl.) is a forest tree species that can sequester large amounts of carbon depending on the silvicultural regime used and the proportion of heartwood present [17], and its natural forests show a positive net carbon balance even under elevated stress conditions caused by drought [18]. Understanding the genotype x environment interaction (GxE) of this species may lead to higher productivity and efficient carbon sequestration when optimal population for a suitable environment is identified.

Coast redwood naturally occurs along a narrow coastal strip (∼50 km width) of western North America ranging from Southern Oregon (OR) in the north to Southern California (CA) in the south [19]. Many sites in its natural range receive little summer precipitation, however, as most of its range occurs in Californian coastal fog belt, fog water is an important source of moisture for this forest ecosystem [20]. One study found that 45 m high adult trees require approximately 600 litres of water per day [20]. Ambrose et al. [21] found that coast redwood invests more resources in producing above-ground biomass than in a robust root system development, and concluded the species is susceptible to drought stress. The related species giant redwood (*Sequoiadendron giganteum* (Lindl.) J.Buchholz) has instead invested into more robust root system on account of reduces above-ground biomass production as a strategy to cope with drought. Coast redwood has few pest and diseases. Parts of redwood trees such as leaves, branchlets, roots and wood are rarely damaged by non-human animals presumably due to the volatile essential oils and tannins content. While there is a number of insects infesting coast redwood trees, none of them is capable of causing the death of mature trees [22]. Coast redwood leaves, branchlets, roots and wood are also notably decay-resistant, likely due to volatile essential oils (monoterpenoids and sesquiterpenoids) and tannins [19].

The objective of this study was to examine the genotype x environment interaction (GxE) of coast redwood by comparing tree growth among a number of different environments across New Zealand. We examined GxE interaction within a genetic framework using a range-wide collection of clones planted across 11 environments [23]. We used broad-sense heritability to assess the proportion of phenotypic variance explained by genotype, and the genetic correlation among sites to examine plasticity for two traits: productivity measured indirectly as stem diameter at breast height (DBH, 1.4 m), and the occurrence of epicormic sprouts (EPI). We also investigated population response regarding environmental parameters at both origins and planted sites through the implementation of Universal response function. The implications of the gained knowledge on where to plant productive coast redwood for carbon are discussed.

## Results

### Detection of population clusters and their correlations with environmental variables

**Fig. 1:**
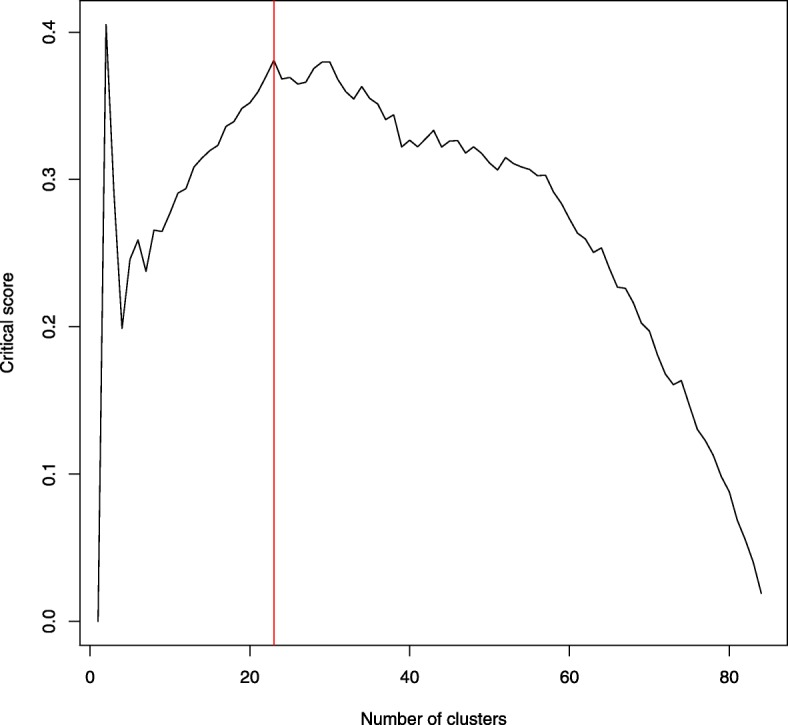
Optimal number of clusters. Optimal number of clusters defined by partitioning around the medoids clustering (PAMK) based on geographical coordinates and climatic variables

**Fig. 2:**
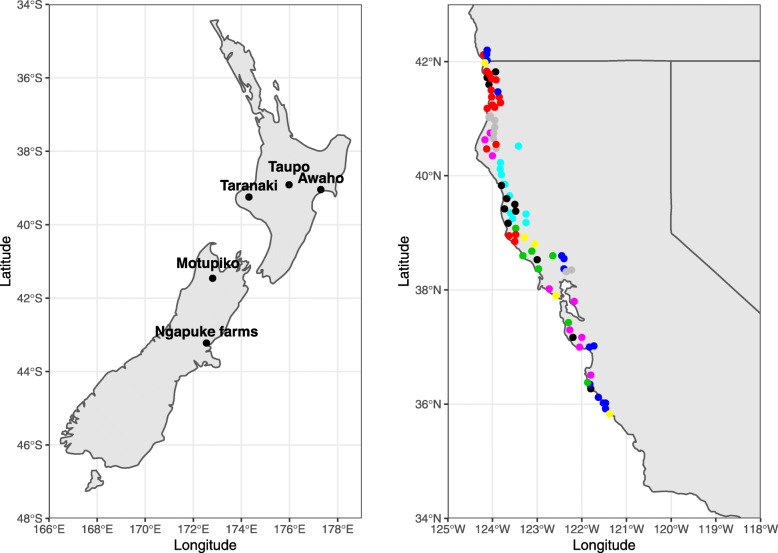
Map of sample distribution. Geographical distribution of sites across New Zealand (left) and distribution of population in coast redwood sample (colours represent population clusters defined by PAMK algorithm) (right) (Figure created in this study using R packages "ggplot2" [24] and "maps" [25])

A cluster analysis based on geographical covariates and climatic variables generated only two significant clusters of genotypes, which were separated by location from the northern and southern parts of the US geographical distribution (data not shown). The second-best scenario defined by critical score detected 23 clusters (Fig. 1), which represented geographically unique and climatically homogeneous regions (Fig. 2 - right plot). The latter scenario was used in the subsequent analysis to obtain a more detailed insight into factors contributing to natural selection and genetic divergence.

### Genetic parameter estimates

An analysis of each of the five sites (Additional file 1: Table S1) found virtually no genetic component at the Taupō site, and this site was therefore removed from all downstream analysis. We hypothesize that the lack of a genetic expression was likely from the significant site limitations on productivity at the Taupō site. These limitations will be discussed further in the discussion.

The multi-site variance component analysis of the four remaining sites generated statistically significant genetic components and heritabilities in both traits analysed. The genetic variance was partitioned into populations and genotype-within-population components, both of which were found to be statistically significant for the two traits (Table 1). The genotypic variance was prevalent in EPI but not in DBH, where differences between populations generally showed a larger proportion of genetic variance compared with the genotype-within-population variance. The population-level broad-sense heritability for DBH ranged from 0.24 to 0.40 while the genotype-level broad-sense heritability ranged from 0.09 to 0.25. The population-level broad-sense heritability for EPI ranged from 0.01 to 0.02 while the genotype-level heritability ranged from 0.09 to 0.18 (Table 1).

**Table 1:** Variance components and broad-sense heritability estimates

Trait	Param.	Awaho	Taranaki	Moupiko	Ngapuke f.
	Pop	0.41 (0.15-0.98)	0.35 (0.06-1.00)	0.24 (0.08-0.66)	0.46 (0.21-1.17)
	Gen	0.35 (0.26-0.49)	0.28 (0.20-0.38)	0.23 (0.15-0.33)	0.15 (0.08-0.23)
	Rep	0.00 (0.00-0.01)	0.00 (0.00-0.01)	0.01 (0.00-0.06)	0.02 (0.00-0.11)
DBH	Rep(Block)	0.00 (0.00-0.01)	0.00 (0.00-0.01)	0.00 (0.00-0.02)	0.00 (0.00-0.02)
	Error	0.65 (0.60-0.72)	0.68 (0.62-0.73)	0.74 (0.68-0.81)	0.64 (0.51-0.85)
	*H*^2^ - Pop.	0.29 (0.14-0.50)	0.30 (0.10-0.53)	0.24 (0.09-0.41)	0.40 (0.22-0.58)
	*H*^2^ - Gen.	0.25 (0.16-0.33)	0.19 (0.12-0.29)	0.18 (0.11-0.26)	0.09 (0.05-0.16)
	Pop	0.02 (0.00-0.07)	0.02 (0.00-0.09)	0.02 (0.00-0.07)	0.01 (0.00-0.12)
	Gen	0.11 (0.06-0.17)	0.08 (0.05-0.14)	0.15 (0.10-0.24)	0.06 (0.11-0.27)
	Rep	0.00 (0.00-0.04)	0.02 (0.00-0.10)	0.02 (0.00-0.10)	0.00 (0.00-0.03)
EPI	Rep(Block)	0.01 (0.00-0.04)	0.01 (0.00-0.04)	0.01 (0.00-0.04)	0.01 (0.00-0.03)
	Error	0.87 (0.79-0.95)	0.87 (0.80-0.95)	0.81 (0.74-0.90)	0.83 (0.75-0.90)
	*H*^2^ - Pop.	0.02 (0.00-0.07)	0.02 (0.00-0.08)	0.01 (0.00-0.07)	0.02 (0.00-0.10)
	*H*^2^ - Gen.	0.11 (0.06-0.16)	0.09 (0.05-0.14)	0.16 (0.10-0.23)	0.18 (0.11-0.25)

Variance components, broad-sense heritabilities and their 95% confidence limits for each site and trait obtained from multi-environment model

### Genotype x environment interaction

**Fig. 3:**
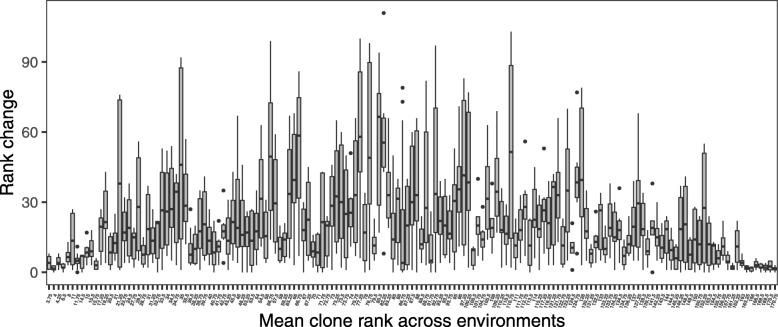
Genotype by environment interaction in DBH. Rank change of the genotype performance for DBH across four environments

**Fig. 4:**
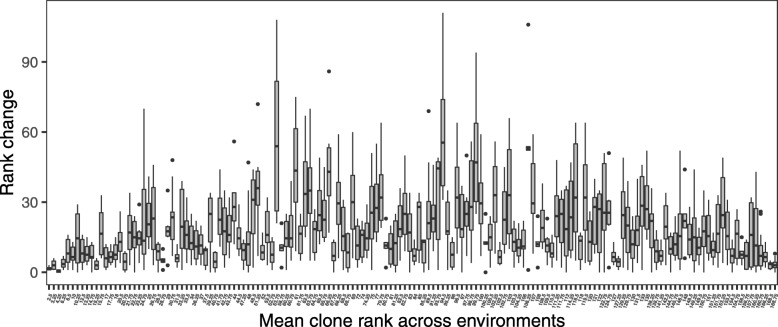
Genotype by environment interaction in EPI. Rank change of the genotype performance for EPI across four environments

Broad-sense genetic correlations between environments (Tables 2 and 3) were used to investigate the interaction between populations and environment (PxE). Important population genetic components were found for DBH, which resulted in high and statistically significant genetic correlations between environments at the population level ranging from 0.98 to 0.99 (Table 2). These results did not indicate any important PxE interaction across the investigated environments at the population level, but the moderate genetic correlations were determined at a genotype level for the same trait, ranging from 0.60 to 0.91 (Table 2). The highest genetic correlation was found between the Awaho and Taranaki sites, confirming the results found in a previous analysis [23]. No statistically significant genetic correlation between sites at the population level was found for EPI, which reflected the negligible population variance components (Table 3 - above diagonal). However, broad-sense genetic correlations between sites at the genotype level were high and were mostly statistically significant (Table 3 - below diagonal). An exception was between the Awaho and Ngapuke farms sites, where the correlation was 0.42 (Table 3). These results indicated mostly only subtle changes in genotype ranking in EPI among sites except for the Awaho and Ngapuke farms sites, which caused large shifts in ranks. Changes in genotype ranking were also investigated, and the performance of some individuals was found to be more stable than others (Figs. 3 and 4), indicating high genotype differences in sensitivity to different environments.

**Table 2:** Genetic correlations for DBH

DBH	Awaho	Taranaki	Motupiko	Ngapuke farms
Awaho	1	0.99 (0.89-0.99)	0.99 (0.91-0.99)	0.99 (0.93-0.99)
Taranaki	0.91 (0.80-0.98)	1	0.99 (0.84-0.99)	0.99 (0.89-0.99)
Motupiko	0.64 (0.43-0.80)	0.66 (0.44-0.81)	1	0.98 (0.90-0.99)
Ngapuke farms	0.75 (0.49-0.92)	0.74 (0.49-0.92)	0.60 (0.31-0.85)	1

Between sites broad-sense genetic correlations at population (above diagonal) and genotype (below diagonal) level and their 95% confidence limits for DBH

**Table 3:** Genetic correlations for EPI

EPI	Awaho	Taranaki	Motupiko	Ngapuke farms
Awaho	1	0.70 (-0.45-0.95)	0.60 (-0.55-0.92)	0.78 (-0.35-0.97)
Taranaki	0.83 (0.54-0.95)	1	0.31 (-0.76-0.86)	0.80 (-0.50-0.97)
Motupiko	0.78 (0.50-0.94)	0.85 (0.55-0.97)	1	0.66 (-0.55-0.95)
Ngapuke farms	0.42 (0.09-0.74)	0.73 (0.44-0.92)	0.80 (0.50-0.94)	1

Between sites broad-sense genetic correlations at population (above diagonal) and genotype (below diagonal) level and their 95% confidence limits for EPI

### Universal response function

**Fig. 5:**
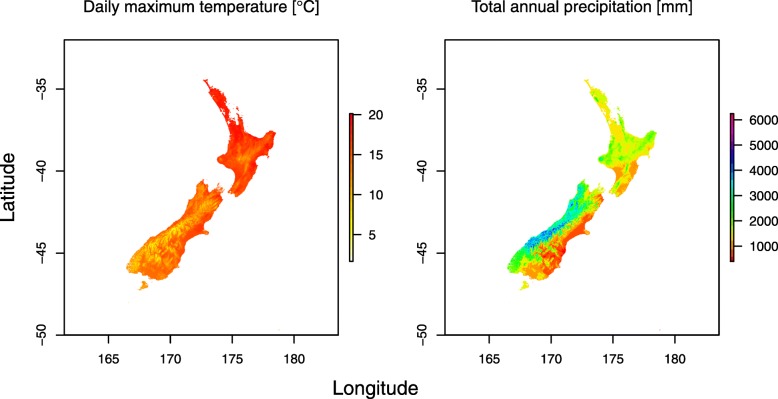
Climate variables distribution across New Zealand. Distribution of climate variables (mean daily maximum temperature - left; total annual precipitation - right) implemented in Universal response function for coast redwood clusters (Figure created in this study using R package "raster" [27])

**Fig. 6:**
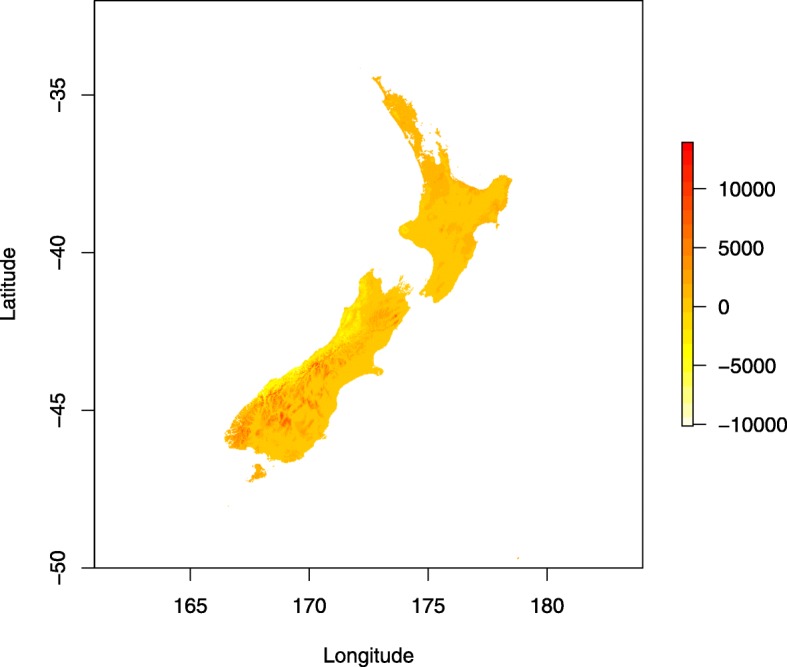
Predicted performance of the genetically best material (Cluster 19) across New Zealand. Predicted DBH [mm] using maximum temperature and precipitation implemented in Universal Response Function (Figure created in this study using R package "raster [27])

We investigated climate variables on the prediction of productivity through Universal response function (URF) [16]. Since the coast redwood occupies relatively uniform environment centered in a fog belt within the Californian coastal area, most of the climatic variables investigated in this study showed high pairwise positive or negative correlations (Table 4) which resulted in URF model singularity. Therefore only two climatic variables with a moderate correlation of -0.55 (Tmax and precipitation) were considered in the final URF model. The proportion of variance explained by the model in term of R^2^ reached 0.972. The statistically most significant variables were Tmax and precipitation at the planted sites in New Zealand as well as their squared terms. The square term of Tmax was only significant climatic variable at origin (Table 5). The highest contribution to the variance explained by the URF model showed Tmax and precipitation at planted sites (Table 5). The URF model was implemented as a tool to predict the performance of the best cluster identified on the basis of quantitative genetic analysis across New Zealand using Tmax and precipitation information (Fig. 5) extracted from WorldClim database [26]. We found reasonable predictability within an area of planted sites (North Island and the north part of South Island) while poor performance was observed outside tested areas in the south of South Island (Fig. 6).

**Table 4:** Correlations between climatic and geographical variables

	Long.	Lat.	Tmax.	Tmin.	Precip.	Radiation	WVP	Wind speed
Long.	1	0.84	0.59	0.57	0.09	0.83	0.52	-0.16
Lat.	0.95	1	0.51	0.68	0.48	0.98	0.75	-0.02
Tmax.	-0.93	-0.93	1	0.92	-0.30	0.60	0.82	0.02
Tmin.	-0.89	-0.95	0.88	1	0.07	0.78	0.97	0.24
Precip.	0.66	0.51	-0.55	-0.62	1	0.48	0.24	0.54
Radiation	-0.96	-0.94	0.91	0.96	-0.75	1	0.83	0.12
WVP	-0.92	-0.98	0.94	0.98	-0.55	0.95	1	0.21
Wind speed	-0.87	-0.90	0.82	0.97	-0.76	0.96	0.93	1

Correlation between climatic variables (Tmax - mean daily maximum temperature, Tmin - mean daily minimum temperature, Precip - annual precipitation, WVP - water vapour pressure) and geographical coordinates (Lat - latitude, Long - longitude) at locations of origin (below diagonals) and at planted sites (above diagonals)

**Table 5:** Universal response function

Variable	Estimate	t-val	P-val	R^2^
Intercept	114 (0.996)	114.98	<0.0001	
Tmax_US	16.59 (12.47)	1.331	0.186197	0.09
Tmax_US^2^	39.57 (11.23)	3.524	0.000634	0.11
Tmax_NZ	-483.94 (27.23)	-17.769	<0.0001	0.95
Tmax_NZ^2^	1146.77 (25.31)	45.312	<0.0001	0.95
Tmax_US*Tmax_NZ	36.22 (114.81)	0.315	0.7531	0.00
Prec_US	22.46 (12.74)	1.763	0.0808	0.01
Prec_US^2^	9.77 (10.93)	0.894	0.3734	0.01
Prec_NZ	-974.91 (27.68)	-35.218	<0.0001	0.88
Prec_NZ^2^	-869.41 (24.82)	-35.03	<0.0001	0.92
Prec_US*Prec_NZ	26.72 (116.40)	0.230	0.8189	0.00
R^2^		0.972		

The results from universal response function using climatic variables: mean daily maximum temperature at origin (Tmax_US), mean daily maximum temperature at planted site (Tmax_NZ), total annual precipitation at origin (Prec_US) and total annual precipitation at planted site (Prec_NZ)

## Discussion

### Genetic background

Extensive natural distributions of forest trees over highly variable environments predispose the adaptive traits of forest trees to exhibit the effects of strong local natural selection. This effect can result in different phenotypic responses from different populations when compared using common garden experiments [28–30]. This is likely to be the case for DBH in coast redwood, which shows a considerable difference in phenotypic expression among populations when tested outside its natural distribution [23] and confirmed across more sites in this study. Interestingly, the differences in total DBH (a measure of productivity) between populations were larger compared to the within-population component (Table 1). This result indicates that natural selection has a strong effect. In contrast, previous studies of indigenous stands of this species in North America using molecular markers found high genetic variation within populations while only subtle variation between populations [31] and only weak and statistically non-significant level of genetic differentiation due to population structure in terms of Wright’s fixation index F _ST_ (0.0031) [32]. These findings correspond to the general notion that growth aligns with forest tree adaptive traits, as growing fast maximises the competitive ability of individual trees for a light interception in a forest [33]. Significant differences in productivity among populations have also been found in other forest tree species, such as poplars [9, 10] and European beech [30] by comparing genetic differentiation of quantitative traits between populations (Q _ST_) and genetic differentiation between populations based on selectively neutral markers (F _ST_) [34, 35]. In this study, diameter at breast height (DBH) was the only attribute representing tree growth. Studies across other forest tree species found strong/moderate correlations between DBH and tree height or volume [36–39] which provides evidence of using DBH as an ideal proxy phenotype for other essential attributes. However, there is decent genetic variability found in DBH - tree height relationship due to differences in individual’s response to environment and competition [40].

Genetic variance and interaction of the genetic and environmental components were investigated at both the population and genotype level. The population-level broad-sense genetic correlations between sites for DBH exceeded 0.7, so the PxE interaction was considered to be non-significant [41]. Since the investigation of genotype by environment (GxE) requires the presence of both genetic and environmental variance, the Taupō site was removed from the analysis due to lack of genetic component. We hypothesize that it was due to the low productivity at the site, which we discuss further below. Similar to our study, the missing genetic component was found for drought tolerance in Norway spruce when tested outside the range of species natural distribution [42]. Some GxE interactions at the genotype-within-population level were found when broad-sense genetic correlations between sites decreased slightly below 0.7 in most cases, except Awaho - Taranaki and Awaho - Ngapuke farms. The existence of a GxE interaction at the genotype level when suboptimal sites were involved in the evaluation is possibly due to the sensitivity of coast redwood growth to inbreeding depression. Inbred individuals may perform well under optimal conditions, but their productivity will decrease relative to outbred individuals under stressed conditions [43–45]. Moreover, thanks to intensive vegetative propagation through epicormic sprouts, coast redwood in natural forest stands have been known for the creation of polycormons up to at least 40 meters [31, 46], highlighting the likelihood of the occurrence of inbreeding. The results of this study suggest that young genotypes with unknown pedigrees should, therefore, be tested in moderately stressed environments to eliminate them for use in pedigreed progeny tests [44].

Some individuals were found to have large changes in ranking across environments compared with others (Figs. 3 and 4). We postulate that these individuals may originate from inbreeding. The molecular marker-based analysis could be used to determine whether or not this is the case. Coast redwood is a spontaneous polyploid, presumably developed through genome duplication [47], which can introduce obstacles to the generation of genomic resources. However, several studies have successfully developed and implemented genetic markers in this species [32, 48]. Current development of high-throughput genotyping technologies [49] can generate a significant number of markers to construct marker-based relationship matrices, even in wild populations [50] and thus allow for the investigation of individual inbreeding coefficients [51]. Besides, this genomic information can be used to infer genetically relevant population structures, which could substitute the cluster analysis and provide a more realistic estimate of population genetic variance, as well as an inference about the adaptive potential of studied traits through comparison of genetic differentiation detected in quantitative traits and neutral genetic markers (Q _ST_ versus F _ST_).

The appearance of epicormic sprouts on trees was found to be strongly influenced by genotype, but this was less obvious when fitting the population component. This pattern was the opposite of that observed for DBH and could imply that the EPI trait is not under natural selection. The production of epicormic sprouts presents a means of regenerating the understorey after wildfires [52] which are unlikely to be confined in such a way that causes strong local natural selection. This reasoning is supported by the fact that the ability to produce epicormic sprouts was observed across the whole sample set. The broad-sense heritability for EPI ranged from 0.08 to 0.19, which was low but consistent with previous studies [23, 53]. The low heritability indicates that environmental factors influence the phenotypic expression more than genetics, or that the main part of genetic variance has been already fixed. An experimental study that focused on the effect of pruning O’Hara and Berrill [54] found that the genetic component diminished a few years after the initiation of epicormic sprouts.

This investigation of the PxE interaction found high but non-significant broad-sense genetic correlations across environments at the population level, which is assumed to be connected to a negligible variance component estimate for the effect of populations. The GxE interaction generally showed stable expression in terms of broad-sense genetic correlations between environments at a genotype level. A strong GxE interaction was found only between the Awaho and Ngapuke farms sites (Table 3). The differences in genotype ranking across these two sites can be related to a different level of phenotypic expression. While the Ngapuke farms site showed the highest broad-sense heritability for EPI, the Awaho site showed the lowest (Table 1). However, the persistence of an estimable genetic component should be re-evaluated when the trees are older [54].

### Environmental cues

The analysis of individual sites showed significant genetic components at four of the five sites examined. Virtually no genetic component was found in either of the studied traits at the Taupō site (Additional file 1: Table S1). Trees at the Taupō site received ∼100 - 350 mm more annual precipitation than the other sites (Table 6) but had sandy pumice soil that would drain rapidly, and the volcanic soils in the region typically have low nutrients [55]. The low water retention of sandy soils can create elevated stress through lack of water accessibility during summer drought. Water accessibility is a significant factor affecting coast redwood growth [23], and fog is an important source of moisture for naturally occurring US coast redwood forest stands [20, 56]. Additionally, the fog has been identified as a significant direct and indirect source of nitrogen to coast redwood trees [57]. The presence of drought stress causes a decrease in stem water potential and increases in the occurrence of stem embolisms, which is more pronounced in coast redwood compared with giant redwood [21]. Interestingly, their study also failed in finding any population differences in growth under stressed conditions; however, they investigated longitudinal rather than coast - inland pattern. We hypothesize that the lack of an estimable genetic component at Taupō could be caused by the fast-drying, free-draining soil which can cause water limitations for coast redwood, perhaps contributed to by the relatively poor rooting system of the species compared with others in the *Sequoia* genus [21]. Larger sized estimates of genetic components/heritability can generally be reached under optimal growing conditions, where the expression of underlying genetics is also optimal or close to optimal. Any sub-optimal conditions such as environmental stress can result in the reduction and potential disappearance of the expression of the underlying genetic potential and the reduction in the estimated genetic component [42, 58]. Hence, it is likely that the conditions at the Taupō site are limiting for the growth of planted coast redwood in New Zealand.

**Table 6:** Description of planted sites

Climate var.	Awaho	Taranaki	Taupō	Motupiko	Ngapuke farms
Soil type	Silt loam	Fine sandy loam	Sand	Silt loam	Hill soil
Rooting [m]	1.2	1.35	0.34	0.82	0.57
Soil order	Recent	Allophanic	Pumice	Brown	Pallic
Tmax.[C^∘^]	16.6	16.5	15.4	15.3	13.9
Tmin [C^∘^]	7.2	7.8	5.5	5.9	4.9
Rain. [mm]	1506	1740	929	1564	2007
Solar rad.	173483	173278	165250	172300	168396
Water vapour press.	1.05	1.16	0.91	0.99	0.90
Wind speed	4.5	4.6	4.1	3.8	4.7

Environmental conditions at investigated sites: soil type, estimated rooting depth, soil order, mean annual rainfall (Rain.), mean daily maximum temperature (Tmax) and mean daily minimum temperature (Tmin)

Our study attempted to identify the climate variables that affect coast redwood productivity in New Zealand through the implementation of the universal response function. However, the potential of universal response function (URF) to achieve this goal was rather limited due to the fact that coast redwood occupies ecologically relatively uniform environment and most of the investigated climate variables were highly correlated (Table 4 - above diagonals). Consequently, the UFR model resulted in the singularity issue when more than two variables were included. Therefore, exploration of co-linearity between variables is required to avoid any singularity in the URF model when species occupies environmentally/geographically narrow space. Our final model contained only two variables that show only moderate correlation (mean daily maximum temperature and total annual precipitation). The limited number of variables included in the URF model can restrict the informativeness of the model to predict species productivity reliably. It is worth to mention at this point that we used climatic data available in WorldClim database [26]. There are other potentially useful resources of climate variables such as FAO agroclimatic databases and mapping tools [59] or NASA’s distributed active archive centres (DAAC) [60].

The implemented URF model indicated the climate variables (mean daily maximum temperature and total annual precipitation) at planted sites as the most significant factors affecting productivity in New Zealand along with the quadratic term of mean daily maximum temperature at the origin. In contrary, an only a weak signal was detected in climate variables at origin (Table 5). The non-significance of climate variables at origin can be related to a relatively small number of tested genotypes per each level of climate variable despite a broader range of magnitudes compared to climate variables at planted sites. Additionally, the environmental variables represent coarse level environmental variables and do not reflect potentially important intra-annual climatic dynamics (e.g. number of dry days between precipitation events, precipitation event duration and intensity), which could be important factors for growth and stress for individual provenances. Research with other tree species has found some genotypes are more sensitive to microsite and climate than others [61]. The dendrochronological study conducted *in situ* on standing Californian coast redwood trees found a latitudinal pattern in climate sensitivity with a strong contrast between cooler northern rainforests and warm and dry southern forests. A negative relationship between radial growth and cloudiness of the local environment was found in trees growing in northern or central latitudes whereas a negative relationship was found between growth and dry summers in trees located at southern latitudes. Cloudiness is thought to reduce the amount of sunlight in reaching the surface of the needles, which reduces photosynthesis and leads to less radial growth [62].

Additionally, total annual precipitation is coarse environmental parameter as it does not reflect the variability in precipitation distribution and intensity throughout the year, which may be more critical for plant adaptation to cope with summer drought. In coast redwood’s natural range, fog water is a significant water source, especially for coastal populations [45]. It has been estimated that fog contributes 25 to 50% of total annual water input for coast redwood ecosystems, and decreases with increasing distance from the ocean [20, 63]. Conversely, New Zealand rarely gets summer fog, let alone the frequency of coastal California. Thus, it is unlikely to be a significant water site for the five sites at any time of the year. Unlike California, the majority of areas in New Zealand regularly receive precipitation throughout the year due to its maritime climate - although less precipitation during summer. It is unknown if New Zealand’s maritime climate and regular precipitation act as an effective substitute for fog water during the summer, and if New Zealand grown redwood is more water-stressed during this period. Anekonda et al. [64] found a strong correlation between canonical variables inferred from growth increment, and respiratory variables, implying respiration may be correlated with growth. Estimates of the efficiency of each individual tree’s energy metabolism showed that highly productive genotypes are less energy-efficient compared with less productive genotypes, something that appears to be contradictory.

The universal response function [16, 65] has already been implemented to predict population performance outside the range of species’ natural distribution in the case of Douglas-fir in Central Europe [66] or white spruce in Canada [67]. The URF developed in this study explained 88% of the variation in data which is very similar to our results. Additionally, the authors found that conditions at planted sites are more significant compared to conditions at population origin, which is similarly found in our study. Chakraborty et al. [66] found the advantage of the empirical models such as URF over the climate envelope models and recommended their implementation, especially in case of productivity predictions outside of species’ natural distribution.

The URF model was implemented to predict the productivity of genetically superior material identified in quantitative genetic analysis (Cluster 19) across New Zealand. The results show the best performance at the north of North Island (Northland) as well as the coastal line along the east coast of North Island. These regions were identified in the previous study using data from permanent sample plots analyse by multiple regression model [68]. However, the predictions outside the range of the tested environments produced unreliable estimates, especially in the southwest of South Island. This can be attributed partially to the lack of tested environments as well as to the limited number of environmental variables included in the URF model. We expect that the inclusion of more tested environments would allow for better estimation of co-linearity between environmental variables at tested sites and thus their better selection for the final model. Therefore, more detailed exploration of genotype x environment interaction through universal response function would require a larger sample size tested across more environments.

## Conclusion

The current study provides evidence of the ability to detect a heritable component of phenotypic variation in productivity, as measured with DBH, and epicormic sprouts in a sample of coast redwood outside its natural conditions. However, also environmental conditions under which genetic component was suppressed were detected. The investigation of genotype x environment interaction did not discover any serious interaction of population/genotype with the environment. However, some combinations of sites showed slight effects in genotype ranking, specifically for particular genotypes which were attributed to possible selfing. Nevertheless, such hypothesis will need to be approved by the implementation of genetic markers. The implementation of universal response function allowed for the identification of important environmental factors affecting species productivity. Additionally, this approach enabled us to predict the performance of species across the New Zealand environmental conditions. In combination with quantitative genetic analysis which identified genetically superior material, the URF model can directly identify the optimal geographical regions to maximize productivity. However, the finding of ideally uncorrelated climatic variables for species with narrow ecological amplitude is rather challenging, which complicates construction of informative URF model. This, along with a small number of tested environments, tended to overfit a prediction model which resulted in extreme predictions in untested environments.

## Methods

### Plant material

Coast redwood material was collected in 1984 from 98 locations along the west coast of USA representing most of its natural distribution, i.e. from Curry County (OR) to Monterey County (CA) (Fig. 1) [23]. Seedlings were collected where possible, with saplings or stump sprouts obtained if no seedlings were available. A total of 198 genotypes representing all 98 different geographical locations were clonally propagated. Of these, a set of 182 genotypes representing 85 locations (1 - 6 genotypes/location (Additional file 1: Table S2)) was imported into New Zealand in 2000, clonally propagated and used to establish a series of clonal trials across New Zealand between 2003 and 2006. A more detailed description of the material can be found in [23]. All individuals were phenotyped for diameter at breast height (DBH) and epicormic sprouts (EPI). Ageing from the different types of material is likely to have differed, but the data on the origin of the cuttings was not available, so this will be confounded with the results.

### Trial sites

Eleven field trials were established, and data from five of these trials having the most complete sample data representation are analysed here. Three sites were in the North Island and two in the South Island of New Zealand (Fig. 2). Details of the sites are given in Table 6. The sites represented a range of environmental conditions. The Awaho and Taranaki sites were situated on fertile soils with abundant precipitation, which corresponded to coast redwood’s optimum growth conditions. The Ngapuke farm site had a much lower annual precipitation and was planted on a northwest slope that dries out in summer. The Taupō site received more precipitation than two of the other sites but had sandy soil. Previous analyses [23] have been undertaken on only two sites. Each genotype at each site was represented by several identical copies (ramets) planted in incomplete block design.

### Climatic variables

Climatic variables such as mean daily minimum temperature [C ^∘^] and mean daily maximum temperature [C ^∘^], annual precipitation [mm], solar radiation [kJ m ^−2^ day ^−1^], water vapour pressure [kPa] and wind speed [m/s] were extracted from WolrdClim database version 2 (http://worldclim.org/version2) [26] as monthly averages across 1970–2000 period. The variables related to temperature, solar radiation and wind speed were averaged across months while precipitation was summed across months for downstream analysis.

### Statistical evaluation

Each location was represented by 1 - 6 trees (Additional file 1: Table S2), so population structure was inferred by using partitioning around the medoids clustering algorithm implemented using the "pamk" function available in the "fpc" R package [69]. The geographical coordinates (latitude, distance from the coast and elevation) and climatic variables (annual precipitation, annual daily temperature maximum and minimum) (Additional file 1: Table S2) were used as inputs to create groups of genotypes from geographically discrete and environmentally homogeneous regions. Genetic parameters, such as genetic variance (including both additive and non-additive components), broad-sense heritability and genetic correlations between different environments were estimated through a multivariate generalised mixed model based on Monte Carlo Markov Chain sampling [70] as follows:

$$ \boldsymbol{Y}=\boldsymbol{X}\boldsymbol{\beta}+\boldsymbol{Zp}+\boldsymbol{Zg}+\boldsymbol{Zr}+\boldsymbol{Zr(b)}+\boldsymbol{e} $$

where ***Y*** is a matrix of phenotypes, ***β*** is the vector of fixed effects (intercept), ***p*** is the vector of random population effects following var(***p***) ∼N(0,G1), where G1 is variance-covariance structure of population effects following G1= $\left [\begin {array}{ccc} \sigma _{p_{1}}^{2} & \hdots & \sigma _{p_{1}p_{n}}\\ \vdots & \ddots & \vdots \\ \sigma _{p_{n}p_{1}} & \hdots & \sigma _{p_{n}}^{2}\\ \end {array}\right ]\bigotimes $***I***, where $\sigma _{p_{1}}^{2}$ and $\sigma _{p_{n}}^{2}$ are population variances for the 1^st^ and n^th^ environment, $\sigma _{p_{n}p_{1}}$ and $\sigma _{p_{1}p_{n}}$ are population covariances between the 1^st^ and n^th^ environment, $\bigotimes $ is the Kronecker product and ***I*** is the identity matrix. The vector ***g*** is vector of random genotype within population effects following var(***g***) ∼N(0,G2), where G2 is variance-covariance structure of genotype within population effects following G2=$\left [\begin {array}{ccc} \sigma _{g_{1}}^{2} & \hdots & \sigma _{g_{1}g_{n}}\\ \vdots & \ddots & \vdots \\ \sigma _{g_{n}g_{1}} & \hdots & \sigma _{g_{n}}^{2}\\ \end {array}\right ]\bigotimes $***I***, where $\sigma _{g_{1}}^{2}$ and $\sigma _{g_{n}}^{2}$ are genotype within population variances for the 1^st^ and n^th^ environment. The vector ***r*** is vector of random replication effects following var(***r***) ∼N(0,G3), where G3 is variance-covariance structure of replication effect following G3=$\left [\begin {array}{ccc} \sigma _{r_{1}}^{2} & \hdots & \sigma _{r_{1}r_{n}}\\ \vdots & \ddots & \vdots \\ \sigma _{r_{n}r_{1}} & \hdots & \sigma _{r_{n}}^{2}\\ \end {array}\right ]\bigotimes $***I***, where $\sigma _{r_{1}}^{2}$ and $\sigma _{r_{n}}^{2}$ are replication variances for the 1st and nth environment. The vector ***r(b)*** is vector of random block nested within replication effects following var(***r(b)***) ∼N(0,G4), where G4 is the variance-covariance structure for block nested within replication effects following G4=$\left [\begin {array}{ccc} \sigma _{r(b)_{1}}^{2} & \hdots & \sigma _{r(b)_{1}r(b)_{n}}\\ \vdots & \ddots & \vdots \\ \sigma _{r(b)_{n}r(b)_{1}} & \hdots & \sigma _{r(b)_{n}}^{2}\\ \end {array}\right ]\bigotimes $***I***, where $\sigma _{r(b)_{1}}^{2}$ and $\sigma _{r(b)_{n}}^{2}$ are block nested within replication variances for the 1^st^ and n^th^ environment. The vector ***e*** is vector of random residuals (effect of ramet within genotype) following var(***e***) ∼N(0,R), where R is residual variance-covariance structure following R=$\left [\begin {array}{ccc} \sigma _{e_{1}}^{2} & \hdots & \sigma _{e_{1}e_{n}}\\ \vdots & \ddots & \vdots \\ \sigma _{e_{n}e_{1}} & \hdots & \sigma _{e_{n}}^{2}\\ \end {array}\right ]\bigotimes $***I***, where $\sigma _{e_{1}}^{2}$ and $\sigma _{e_{n}}^{2}$ are residual variances for the 1^st^ and n^th^ environments, ***X*** and ***Z*** are the incidence matrices assigning fixed and random effects to measurements in matrix ***Y***. Broad-sense heritability at population level was estimated as follows:

$$H_{p}^{2}=\frac{\sigma_{p}^{2}}{\sigma_{p}^{2}+\sigma_{g}^{2}+\sigma_{e}^{2}} $$

where $\sigma _{p}^{2}$ is posterior mode of population variance, $\sigma _{g}^{2}$ is posterior mode of genotype variance and $\sigma _{e}^{2}$ is posterior mode of residual variance. Similarly, the broad-sense heritability at genotype level was estimated as follows:

$$H_{g}^{2}=\frac{\sigma_{g}^{2}}{\sigma_{p}^{2}+\sigma_{g}^{2}+\sigma_{e}^{2}} $$

The broad-sense genetic correlation between sites at the population level to define population x environments interaction was estimated using Pearson product-moment estimate as follows:

$$r_{GE_{p}}=\frac{\sigma_{p_{i}p_{j}}}{\sqrt{\sigma_{p_{i}}^{2}\sigma_{p_{j}}^{2}}} $$

where $\sigma _{p_{i}p_{j}}$ is posterior mode of population covariance between i^th^ and j^th^ environment, $\sigma _{p_{i}}^{2}$ and $\sigma _{p_{j}}^{2}$ are posterior modes of population variance at environments i and j, respectively. Similarly, the broad-sense genetic correlation between sites at genotype level to define genotype x environment interaction was estimates as follows:

$$r_{GE_{g}}=\frac{\sigma_{g_{i}g_{j}}}{\sqrt{\sigma_{g_{i}}^{2}\sigma_{g_{j}}^{2}}} $$

where $\sigma _{g_{i}g_{j}}$ is posterior mode of genotype covariance between i^th^ and j^th^ environment, $\sigma _{g_{i}}^{2}$ and $\sigma _{g_{j}}^{2}$ are posterior modes of genotype variance at environments i and j, respectively. All analyses were performed in R ’base’ package [71] with implementation of ’MCMCglmm’ package [70]. The phenotypic data were standardised to have mean 0 and variance 1 [72]. The number of iterations was set to 500,000 and burn-in period was 50,000 runs. Thinning was set to 10.

The graphical investigation of GxE at the individual level was investigated through the construction of boxplots. The individuals were ranked in each environment regarding their performance, and average clonal ranking was estimated as arithmetical mean of genotype ranks across investigated environments. Box of each genotype then represents the dispersion of pair-wise change in rankings between environments. In this way, we can investigate behaving of individuals generally ranked as the best, poor or average.

The universal response function was constructed by using climatic variables at origin as well as at planted sites following [16]:

$$Y_{ij}=b_{0}+b_{1}X_{1i}+b_{2}X^{2}_{1i}+b_{3}X_{2j}+b_{4}X^{2}_{2j}+b_{5}X_{1i}X_{2j}+e_{ij} $$

## Supplementary information

**Additional file 1** Supplemental tables. The Additional file 1 contains Table S1 summarizing variance components and broad-sense heritability from single site analysis and Table S2 describing climatic and geographical variables related to each sampling site in US.

**Additional file 2** Phenotypic data. The Additional file 2 contains all phenotypic data used in this study.

## Data Availability

All phenotypic data and used in the study is available in Additional file 2.

## Abbreviations

CA: California
DBH: Diameter at breast height
EPI: Epicormic sprouts
GxE: Genotype by environment interaction
Lat: Latitude
Long: Longitude
OR: Oregon
Prec_NZ: Total annual precipitation at planted site
Prec_US: Total annual precipitation at origin
PxE: Phenotype by environment interaction
Tmax_NZ: Mean daily maximum temperature at planted site
Tmax_US: Mean daily maximum temperature at origin
Tmin: Mean daily minimum temperature
URF: Universal response function
US: The United States of America
WVP: Water vapour pressure
